# Institutional trauma, absence of accountability, and psychopathology in a juvenile serial homicide case

**DOI:** 10.3389/fpsyt.2026.1855731

**Published:** 2026-05-25

**Authors:** Carlos Ramos-Galarza, Jennifer Obregón, Brenda Guerrero, Jorge Cruz-Cárdenas

**Affiliations:** 1 Centro de Investigación ESTec, Facultad de Psicología, Universidad Indoamérica., Quito, Ecuador; 2 Centro de Atención en Psicología Clínica., Quito, Ecuador; 3 East London Foundation Trust – NHS, London, United Kingdom; 4 Centro de Investigación ESTec, Facultad de Administración y Negocios, Universidad Indoamérica., Quito, Ecuador

**Keywords:** accountability, childhood maltreatment, institutional trauma, Juan Fernando Hermosa, juvenile delinquency, juvenile justice, psychopathology, residential youth care

## Abstract

Juan Fernando Hermosa, infamously known as “The Child of Terror,” committed 23 murders in Ecuador between 1991 and 1992. This perspective article offers a conceptual and theoretical analysis of the interplay between institutional trauma, accountability, and psychopathology in juvenile justice settings, using Hermosa’s trajectory as an illustrative example. Drawing on publicly available judicial records, academic literature, and journalistic sources, we advance the argument that institutional trauma, harm inflicted or exacerbated by juvenile justice and residential systems themselves, and the complete absence of organizational accountability may interact to worsen, rather than reduce, severe antisocial outcomes. Hermosa’s incarceration at the Virgilio Guerrero Institute is examined not as empirical evidence but as a theoretically informative case that highlights the potential consequences of punitive, non-rehabilitative environments. We propose that without procedural, therapeutic, restorative, and systemic accountability mechanisms, juvenile justice settings risk becoming trauma-generating institutions that compound pre-existing psychopathology. Particular attention is given to the hypothesized intersection of early childhood maltreatment and later institutional trauma as a pathway toward severe antisocial development. This perspective concludes by outlining key accountability mechanisms, external oversight, youth grievance procedures, trauma-informed staff training, and transparent error remediation, as essential components for preventing the escalation of violence among at-risk youth. The article aims to stimulate theoretical discussion and inform policy reform rather than to generalize from a single case.

## Introduction

Juan Fernando Hermosa, known as “The Child of Terror”, was a Latin American murderer held accountable for killing 23 people in 4 months. In the present case study, we analyze his personal story, characterized by early exposure to adversity, behavioral disturbances, and escalating patterns of violence. The examination encompasses his modus operandi, victim profile, and psychological traits, with a particular focus on characteristics indicative of psychopathy. Emphasis is placed on the hypothesized influence of childhood maltreatment and neglect on his neurodevelopmental processes and subsequent antisocial trajectory, as well as on his documented history of animal cruelty, which has been correlated in existing literature with later violent and homicidal tendencies.

Beyond the individual level, this case is situated within its historical, social, and economic context, acknowledging that structural conditions, such as poverty, social inequality, and institutional deficiencies, may have contributed to the likelihood of violent outcomes. By adopting a multilevel analytical framework, this report underscores the importance of examining both proximal risk factors (e.g., psychological vulnerabilities, familial adversity) and distal structural determinants (e.g., marginalization, inadequate social control mechanisms). Finally, implications for prevention and intervention are addressed, emphasizing the need for early identification of antisocial tendencies, the implementation of evidence-based psychological interventions, and the strengthening of social support networks to reduce the likelihood of similar outcomes, particularly in comparable contexts.

This paper responds directly to the special issue on Institutional Trauma, Accountability, and Psychopathology in Residential and Juvenile-Justice Youth by examining a case where all three constructs converged tragically. Importantly, this article is written as a Perspective piece rather than a traditional case study. Given that Juan Fernando Hermosa is deceased and no direct psychological assessment, clinical interview, or standardized testing was possible, it is impossible to meet the methodological standards required for a classical case study (e.g., prospective data collection, validated psychometric instruments, or direct patient–clinician interaction). Consequently, we have adopted the format and characteristics of a Perspective article as defined by Frontiers, prioritizing theoretical argumentation, conceptual analysis, and illustrative use of historical documentary sources over empirical generalization. While Hermosa’s early childhood abuse has been well documented, less attention has been paid to the institutional trauma he experienced after incarceration at the Virgilio Guerrero Institute, a juvenile correctional facility characterized by neglect, lack of psychological services, and punitive rather than rehabilitative practices. Furthermore, the concept of accountability, whether the institution acknowledged its failures, provided redress, or implemented corrective measures, was entirely absent. This case thus serves as a critical counterexample: when institutional trauma goes unacknowledged and unaccounted for, psychopathology may worsen, and violent outcomes become more likely. By analyzing Hermosa’s trajectory through this tripartite lens, we aim to demonstrate why accountability must become a central focus of juvenile justice reform.

## The preamble of the crime: the method followed

This article adopts a perspective analysis to examine how specific factors may have influenced the criminal behavior of Juan Fernando Hermosa. Rather than reconstructing his life trajectory through a traditional case study design, we analyze four interconnected domains that emerge from the documentary evidence as potentially significant in his developmental pathway toward extreme violence. First, we examine his psychological profile, including documented traits such as lack of remorse, impulsivity, manipulativeness, aggressive reactions to perceived disrespect, and early animal cruelty, behaviors that theoretical frameworks associate with callous, unemotional traits and externalization of blame (1–3). Second, we analyze childhood abuse, specifically the severe corporal punishment and emotional neglect Hermosa experienced within his adoptive family, and how such adverse experiences may have normalized aggression and disrupted emotional regulation (4–6). Third, we consider contextual factors, including poverty, social marginalization, and institutional weaknesses in Ecuador during the 1980s and 1990s, which limited opportunities for early intervention and reinforced antisocial pathways (7–9). Fourth, we explore the absence of prevention or effective treatment, noting that despite clear warning signs (animal cruelty, theft, gang formation), no evidence-based psychological interventions, family support programs, or rehabilitative services were provided to Hermosa during his development (10–12). By integrating judicial records, academic literature, and journalistic sources, this perspective analysis advances a theoretically informed interpretation of how these four factors may have converged to shape Hermosa’s criminal trajectory, with the goal of stimulating discussion about prevention and accountability in juvenile justice contexts.

## Sources of information

The data were obtained from multiple documentary sources, including:

1. Judicial and police records, which provided information on Hermosa’s criminal acts, arrest, and judicial processes.
2. Academic works, such as articles and books, that analyze his biography and psychological profile.
3. Journalistic sources, including national newspapers, documentaries, and interviews produced between 1991 and 2023. The inclusion criteria required that the selected sources came from a verifiable origin, offered direct or contextualized information about Hermosa’s biography, crimes, or psychological development, and were published by recognized institutions or authors.

Excluded from the analysis were anecdotal reports, speculative commentaries, or sources lacking sufficient reliability.

## Analytic strategy

The analysis was carried out through a documentary review with thematic coding. First, all the collected sources were systematically read and classified, such as biography, modus operandi, victim profile, psychological characteristics, childhood abuse, and social context. Second, recurrent patterns were identified within each category, allowing for the triangulation of information across different types of sources. This process ensured consistency between the descriptive narrative and the evidence reported in the literature.

To enhance methodological rigor, the documentary corpus was examined through iterative thematic analysis following principles of qualitative content analysis. Sources were independently reviewed by two members of the research team, and thematic categories were refined through consensual validation meetings. When discrepancies in interpretation emerged, these were resolved through re-examination of primary source material to ensure interpretive coherence. Although triangulation relied exclusively on secondary documentation, convergence across judicial records, journalistic archives, and academic sources was used as a credibility strategy. Given the retrospective and documentary nature of the data, analytical validation was limited to internal consistency and cross-source corroboration rather than empirical verification.

## Ethical considerations

Given the retrospective nature of the study, which relied exclusively on secondary sources, no direct contact with participants was required. Nevertheless, the analysis adhered to the ethical principles of forensic research, including respect for the dignity of victims, sensitivity in describing violent acts, and avoidance of sensationalism. The objective was to contextualize Hermosa’s case for academic and preventive purposes, rather than to reproduce narratives that could trivialize or glorify criminal behavior.

## Study objectives

The methodological approach directly supports the objectives of this perspective article: (a) to reconstruct the biography and criminal behavior of Juan Fernando Hermosa from a forensic psychology perspective, (b) to analyze the individual, familial, and contextual risk factors that influenced his development, (c) to discuss the implications of this case for prevention and intervention strategies in juvenile delinquency and serial homicide, and (d) to analyze how institutional trauma experienced at the Virgilio Guerrero Institute, compounded by the absence of systemic accountability, may have contributed to Hermosa’s worsening psychopathology and eventual death. By combining diverse documentary sources within a structured analytical framework, the study provides a scientifically grounded narrative that contributes to the understanding of severe antisocial behavior in Latin America.

## The Child of Terror: results of this case construction

### The story behind juvenile criminal activity

Juan Fernando Hermosa, born on February 28, 1976, in Shushufindi, Ecuador, gained notoriety as “The Child of Terror” due to his involvement in a series of violent crimes, including the murder of 23 individuals between 1991 and 1992. Raised in a low-income household with precarious living conditions, Hermosa was placed for adoption at the age of one by his biological mother. The Hermosa-Suárez family adopted him and subsequently relocated to Quito. Despite this change, he experienced emotional neglect and abuse, particularly from his adoptive father, who was frequently absent due to his economic activities and admitted to hitting him as a punishment for his misbehaviors (13, 14).

At an early age, Hermosa learned from a relative that he had been adopted, an event that, according to his adoptive father, led to a marked and troubling shift in his behavior (15). By the age of seven, he exhibited early signs of violence by killing domestic animals. By then, his criminal behavior had escalated to theft, assault, and gambling. At twelve, he formed a gang, and by fifteen, he had begun targeting mainly taxi drivers and homosexual men in a four-month killing period.

In 1992, Hermosa was apprehended by law enforcement following a coordinated operation involving twenty police officers. Upon entering the residence, officers immediately opened fire on a figure lying on a bed, discharging their weapons eleven times. Tragically, the individual was Hermosa’s mother, who was deaf and unable to respond to verbal warnings. By the time of the raid, Hermosa had already fled the premises; however, he was eventually captured during his attempted escape (14). He was detained at the Virgilio Guerrero Institute, a juvenile correctional facility, from which he later escaped, killing a police officer during his flight. Hermosa died under suspicious circumstances in 1996 in Shushufindi; his death is widely believed to have been a homicide (13).

### Modus operandi and victim profile

The present section outlines the behavioral patterns and victim characteristics associated with Juan Fernando Hermosa. His case offers critical insights into the forensic classification of serial homicide and contributes to the literature on the psychological and behavioral development of violent juvenile offenders. The details of his modus operandi are shown in **Table 1**.

**Table 1:** Victim Categories of Juan Fernando Hermosa (November 1991–January 1992).

Victim category	Number of victims	Notes
Taxi drivers	9	Targeted during routine work
Homosexual men	10	Selected as part of the homophobic attacks
Lorry drivers and assistants	3	Killed while working on transport routes
Police officer	1	Killed during Hermosa’s escape from juvenile detention
Total	23	All victims were murdered within approximately four months

All victims were adult males, and most attacks occurred at night in peripheral or rural areas surrounding Quito, Ecuador, specifically in locations such as Calderón, Pintag, and Carapungo. The murders were highly consistent in execution, with victims typically killed by two shots to the head using a 9mm firearm. Forensic evidence later confirmed that all bullets were fired from the same weapon (13, 16).

Hermosa’s criminal behavior was characterized by a distinct modus operandi that developed in both precision and brutality over time. His first recorded homicide occurred on November 22, 1991, when, at the age of 15, he fatally shot a taxi driver inside a San Remo model vehicle after spending the evening with members of his youth gang. The victim’s body was later abandoned on the outskirts of Quito. In the weeks that followed, Hermosa continued to target taxi drivers, selectively choosing vehicles that facilitated more straightforward navigation through the city’s hilly terrain (16, 17).

Hermosa gradually expanded his target pool to include homosexual men. In one instance, he shot a local hairdresser, an acquaintance, after being invited to the victim’s apartment (18). Later, lorry drivers were attacked, and a message was left on one victim’s body that read: “First it was the taxi drivers, then the homosexual men, now the lorry drivers … the police officers have to take care not to be the next ones” (17).

The crimes predominantly occurred during night time hours and weekends. Hermosa often committed these acts alongside members of his gang, though he maintained leadership and direct control over the executions. The killings ceased following his arrest on January 9, 1992.

## Targeting of homosexual men: a specific pattern of victim selection

Beyond the descriptive enumeration of victim categories, the overrepresentation of homosexual men among Hermosa’s victims (10 out of 23) demands specific analytical attention. Unlike taxi and lorry drivers, whose victimization appears predominantly situational or opportunistic, determined by routine occupational exposure and environmental accessibility, homosexual men were likely selected based on a distinct motivational substrate. Forensic and criminological literature has consistently documented that perpetrators who target sexual minorities often act upon a combination of homophobic bias, perceived vulnerability of the victim, and internal conflicts related to masculinity or sexuality (19, 20). In Hermosa’s case, journalistic sources indicate that he frequented areas known for male homosexual encounters and used deception to gain victims’ trust before killing them (21). This pattern suggests that victim selection was not merely opportunistic but reflected a targeted, bias-driven component. Research on hate-motivated homicides indicates that perpetrators often externalize their own insecurities, experience intense shame or anger toward non-normative sexualities, and seek to reassert a threatened masculine identity through lethal violence (22). While a definitive psychological autopsy is impossible given Hermosa’s death, the disproportionate targeting of homosexual men should not be dismissed as incidental; rather, it invites consideration of how homophobia, internalized stigma, and identity threats may intersect with childhood trauma and institutional neglect to shape victim selection in juvenile serial homicide. Future research on similarly situated offenders should systematically assess sexual bias as a potential driver of victim choice.

## Psychological profile of a serial killer

Hermosa exhibited a constellation of behavioral traits that, from a theoretical standpoint, are frequently discussed in the literature on emerging psychopathic and severe antisocial trajectories in juveniles. Early manifestations of violence, including the torture and killing of domestic animals, have been consistently associated with developmental pathways characterized by emotional dysregulation, trauma exposure, and deficits in empathy (1). Such behaviors are often interpreted within developmental psychopathology as early indicators of callous–unemotional traits and persistent conduct problems (2).

Historical accounts also describe a marked lack of remorse, impulsivity, manipulative interpersonal style, and aggressive reactions to perceived disrespect. Within contemporary theoretical models, these features are conceptualized as part of a multidimensional construct encompassing affective detachment, interpersonal dominance, behavioral disinhibition, and chronic antisocial conduct. These dimensions are not understood as isolated traits but as interacting components within broader developmental systems shaped by both individual vulnerabilities and adverse environmental conditions. Hermosa’s *post hoc* rationalizations for the murders, such as attributing his actions to provocation or disrespect, can be interpreted through frameworks of cognitive distortion and externalization of blame, mechanisms frequently described in severe antisocial populations (3).

Moreover, the case fulfills widely accepted criminological criteria for serial homicide. Hermosa killed multiple victims over an extended period of approximately four months. The crimes occurred in discrete episodes with apparent cooling-off intervals between them. The murders demonstrated behavioral consistency, including the repeated use of the same firearm, a similar execution method (two shots to the head), and patterned victim selection targeting specific occupational and social groups. Such behavioral regularities are characteristic of serial homicide typologies described in the forensic literature, including those involving adolescent perpetrators (23, 24).

Taken together, the documented behaviors suggest a developmental trajectory compatible, at a theoretical level, with severe and persistent antisocial pathology emerging in adolescence. However, given the absence of direct clinical evaluation, these interpretations remain inferential and should be understood as theoretically grounded hypotheses rather than definitive diagnostic conclusions. The case illustrates how early trauma, behavioral dysregulation, and escalating violence may converge within a broader ecological and structural context to produce extreme criminal outcomes.

## The influence of childhood abuse on Juan Fernando Hermosa

“When I punished him, I beat him until his buttocks and ribs turned green,” stated Hermosa’s stepfather (14) when describing his methods of disciplining Hermosa during childhood. Such acts of physical punishment were repeatedly inflicted by his father as well, within a broader familial context where corporal punishment was regarded as an acceptable and legitimate means of correcting undesirable childhood behavior (4).

The repeated use of severe corporal punishment within Hermosa’s family environment underscores the role of early adverse experiences in shaping maladaptive emotional and behavioral patterns. Exposure to such violence not only normalizes aggression as a conflict-resolution strategy but may also contribute to the development of emotional dysregulation, attachment disturbances, and antisocial tendencies (5).

Contemporary developmental psychopathology frameworks emphasize the interaction between individual vulnerabilities and environmental risk factors, highlighting how childhood maltreatment can exacerbate the progression toward violent and criminal outcomes (6).

The consequences of childhood maltreatment extend beyond the emergence of antisocial behavior; they can also result in significant alterations in brain functioning. Chronic exposure to early trauma and stress has been shown to disrupt neurodevelopment, particularly in regions such as the amygdala, prefrontal cortex, and hippocampus, areas essential for emotional regulation, impulse control, and moral reasoning (25). In the case of Hermosa, these neurobiological impairments may have contributed to a diminished capacity for empathy, reduced behavioral inhibition, and poor social judgment, all of which are associated with increased risk for violent and criminal behavior.

## Contextual factors

Although Juan Fernando Hermosa’s violent acts were extreme, it is important to situate them within the broader social, familial, and structural conditions that shaped his development. Research on the etiology of juvenile delinquency emphasizes that exposure to chronic childhood maltreatment, family violence, and social marginalization substantially increases the risk of later violent offending (7, 8).

Hermosa’s history of severe corporal punishment, emotional neglect, and early exposure to crime reflects well-documented pathways linking adverse childhood experiences to conduct problems and antisocial trajectories (10, 11). Moreover, systemic issues such as poverty, weak institutional safeguards, and inadequate psychosocial support in Ecuador during the 1980s and 1990s may have exacerbated his vulnerability, limiting opportunities for prevention or rehabilitation. Viewing Hermosa’s case through this lens highlights the need to consider structural and environmental factors, rather than attributing sole responsibility to the individual.

In Ecuador, the decades of the 1980s and 1990s were marked by what many observers describe as “lost decades” in terms of social development. Economic stagnation, recurrent political crises, and structural inequality deepened social exclusion, particularly among children and adolescents (9). Inflation, unemployment, and institutional instability culminated in the 1999 financial collapse, which rapidly increased poverty rates and triggered mass migration. Within this context, public policies towards youth were influenced by international agendas, particularly the United Nations’ framing of young people as “strategic actors for development.” However, Ecuadorian initiatives were often underfunded, fragmented, and more rhetorical than substantive (26).

For young people, this meant growing up in a contradictory environment: on the one hand, they were heralded as key to national development; on the other, they faced high rates of school dropout, child labor, and violence in both domestic and institutional settings. Punitive responses to youth in conflict with the law were common, while systemic investments in education, employment, and psychosocial support remained limited (27). These conditions created a generation of adolescents whose opportunities were sharply constrained by structural poverty and neglect. Within this precarious landscape, cases such as Hermosa’s can be better understood not only as individual pathology but as the tragic intersection of trauma, exclusion, and institutional weakness.

## Prevention of potential similar cases

The following considerations should be understood as theoretically derived implications rather than empirically tested outcomes of the present analysis. While the documentary reconstruction of Hermosa’s trajectory does not permit causal inference, the convergence between his documented risk factors and established developmental criminology models allows for a conceptual discussion of prevention strategies supported by the broader empirical literature.

The prevention of antisocial behavior during childhood and adolescence, particularly among at-risk youth, requires the implementation of evidence-based programs that strengthen protective factors and promote collaboration among parents, peers, and educational institutions. As evidenced in this case, key risk factors contributing to antisocial development include psychological vulnerabilities, such as impulsivity, heightened emotional reactivity, deficits in emotional regulation, and a propensity for risk-taking. In addition, family-related adversities, such as inadequate parental monitoring, exposure to maltreatment, and the use of punitive disciplinary strategies, further exacerbate the likelihood of antisocial outcomes (10, 28).

Conversely, strong social support networks represent a central protective factor. Empirical evidence underscores the role of engaged parenting as a buffer against juvenile antisocial behavior, with pronounced benefits in disadvantaged neighborhoods (28, 29). For instance, when communities explicitly disapprove of antisocial conduct, parents and peers are more likely to identify early warning signs of problem behavior (30). Within school environments, preventive strategies should include counseling services that aim to enhance future orientation and foster a commitment to education, as well as promote prosocial extracurricular activities. Bimodal programs that integrate parent training with school-based social skills development have demonstrated sustained reductions in delinquency through mid-adolescence (12, 31, 32).

Research further highlights the mediating role of peer groups in shaping developmental trajectories, whereby affiliations with prosocial peers foster the acquisition of adaptive social skills and predict declines in offending behavior (33, 34).

Intervention initiatives that incorporate factors such as conflict resolution training, parent-focused interventions to strengthen family relationships, and improve disciplinary practices, have all shown efficacy in mitigating disruptive behavior. These psychological treatments are further described in the following section.

## Proposed psychological treatment for serial killers

Given the developmental trajectory of Juan Fernando Hermosa, marked by early trauma, emotional neglect, and escalating antisocial behavior, it is essential to consider the psychological processes that might have altered his violent path. Although Hermosa’s life ended prematurely, his case provides a critical framework for reflecting on the types of therapeutic interventions that could be applied not only to individuals like him but also to other serial killers who remain alive and may still benefit from structured psychological treatment. By examining evidence-based approaches, this section aims to outline intervention strategies that address both the individual psychopathology and the broader psychosocial contexts that contribute to serial homicide.

The psychological treatment of serial killers presents a unique challenge due to the profound severity of their psychopathology and the entrenched nature of their violent behaviors. While each case must be evaluated individually, standard features such as childhood trauma, antisocial tendencies, callous–unemotional traits, and cognitive distortions allow for the design of therapeutic frameworks that can be adapted across cases.

A central component of treatment is Cognitive Behavioral Therapy (CBT), which has shown consistent efficacy in modifying distorted thought patterns, reducing impulsivity, and addressing externalization of blame. For serial killers, CBT techniques can be tailored to promote anger management, problem-solving, and the recognition of victim perspectives. This approach not only reduces immediate risk behaviors but also provides offenders with practical coping strategies to replace violent responses (35).

Trauma-focused (TF) interventions are also critical, as many serial killers, including Juan Fernando Hermosa, exhibit histories of early abuse, neglect, or exposure to violence. TF-CBT and related therapies target unresolved traumatic experiences that often manifest as emotional dysregulation, dissociation, or compulsive aggression. By addressing these root causes, treatment fosters greater emotional stability and reduces the compulsive drive toward violent expression (36).

In addition, empathy training and moral reasoning interventions play a pivotal role in counteracting the pervasive callousness observed in many serial offenders. Structured programs that emphasize victim awareness, restorative justice, and roleplaying can help individuals develop rudimentary empathic responses and reconsider entrenched moral disengagement. While the restoration of complete empathy may be unrealistic in some cases, even partial gains can reduce dehumanization and promote prosocial decision-making (37, 38).

Group-based therapeutic strategies, such as Aggression Replacement Training or Multisystemic Therapy, can further enhance treatment outcomes. These approaches disrupt antisocial reinforcement within peer networks, provide structured opportunities to practice prosocial behaviors, and integrate family and community systems into rehabilitation. Although serial killers often operate in isolation, interventions targeting broader relational dynamics remain crucial for reducing recidivism and enhancing long term outcomes (39–41).

Finally, treatment must extend beyond the clinical domain to encompass comprehensive rehabilitation, including educational and vocational training, family therapy, and structured re-entry programs. Isolated psychotherapeutic interventions are insufficient in addressing the multifaceted risks posed by serial offenders; instead, systemic approaches that restore social capital and provide alternatives to violent behavior are essential (42).

### Institutional trauma and absence of accountability at the Virgilio Guerrero Institute

Beyond the family and structural adversities described above, Hermosa’s incarceration at the Virgilio Guerrero Institute represents a critical but previously underexplored source of institutional trauma. According to judicial and journalistic records, the facility provided minimal psychological or educational services. Hermosa was confined with older, more entrenched offenders, exposing him to further violence and criminal modeling (13, 16). No evidence exists of trauma-informed care, individual therapy, or family reunification planning. Instead, the institution operated on a punitive logic consistent with Ecuador’s broader juvenile justice approach at the time, which emphasized containment over rehabilitation (9, 26).

Contemporary developmental psychopathology frameworks emphasize that exposure to institutional violence and neglect can produce neurobiological alterations similar to those observed in childhood maltreatment. Chronic exposure to early trauma and stress has been shown to disrupt neurodevelopment, particularly in regions such as the amygdala, prefrontal cortex, and hippocampus, areas essential for emotional regulation, impulse control, and moral reasoning (25). In Hermosa’s case, institutional trauma at the Virgilio Guerrero Institute may have compounded pre-existing neurodevelopmental vulnerabilities stemming from his early childhood abuse, creating a cascade of worsening behavioral dysregulation.

Compounding these neurodevelopmental and psychological consequences described above, the Virgilio Guerrero Institute exemplifies a broader and deeply troubling phenomenon observed across Latin American juvenile justice systems: the transformation of rehabilitation centers into “schools of crime”. Rather than interrupting antisocial trajectories, such facilities often provide a structured environment in which young offenders refine their criminal skills, expand their antisocial networks, and adopt more sophisticated and violent modus operandi (13, 16). In Hermosa’s case, incarceration did not reduce his violent capacity; instead, it was during his detention that he planned and executed his escape, during which he killed a police officer, an act that demonstrated enhanced tactical planning and lethal efficiency compared to his earlier offenses (14, 17). This pattern is consistent with criminological research on prisonization and criminogenic social environments, which demonstrate that prolonged exposure to correctional settings with high concentrations of antisocial peers, weak supervision, and absent rehabilitative programming can increase, rather than decrease, recidivism and offense severity (27, 43).

The problem is particularly acute in Latin America, where juvenile detention facilities are frequently underfunded, overcrowded, and staffed by personnel with limited training in adolescent development or trauma-informed care (9, 26). Instead of providing education, vocational training, psychological therapy, or family reintegration support, interventions known to reduce reoffending, many institutions operate under a punitive logic that prioritizes containment over correction (7, 8). Within such environments, younger or less experienced offenders like Hermosa learn from older, more entrenched delinquents not only specific criminal techniques (e.g., weapon use, evasion strategies, victim selection) but also cognitive distortions that justify violence and externalize blame (3, 43). This process of antisocial modeling has been well documented in developmental criminology: when youth are confined with high-risk peers in the absence of prosocial counterweights, the probability of future serious offending increases substantially (27, 34).

For Hermosa, the Virgilio Guerrero Institute appears to have functioned precisely as such a criminogenic school. Detained at age fifteen with limited prior institutional experience, he was placed among older offenders who had committed more serious and varied crimes (13, 16). Upon escaping, he immediately killed a police officer, a victim type he had not previously targeted, suggesting that his detention may have provided opportunities for learning and skill consolidation (14, 17). Tragically, when Hermosa was later recaptured and died under suspicious circumstances in 1996, any possibility of rehabilitation had long been foreclosed. This trajectory illustrates a core failure of punitive juvenile justice: instead of rehabilitating young offenders and restoring their social opportunities upon release, such systems often return to society individuals who are more dangerous, more skilled in criminal behavior, and less capable of prosocial integration (10, 11, 43).

The implications for the special issue on institutional trauma, accountability, and psychopathology are direct. When juvenile justice facilities operate as schools of crime, they generate institutional trauma not only through direct maltreatment (e.g., violence, neglect) but also through structural design that systematically exposes youth to criminogenic peer influences while withholding evidence-based rehabilitation. Accountability mechanisms, external oversight, transparent outcome monitoring, youth grievance procedures, and staff training in trauma-informed, developmentally appropriate care, are essential to disrupt this cycle (39, 40). Without such accountability, Latin American juvenile justice systems risk perpetuating the very violence they are mandated to prevent, producing graduates of criminal schools rather than rehabilitated young citizens.

## Theoretical hypotheses generated by this perspective

Unlike a traditional case study that aims to describe a single subject in depth, this Perspective article uses Hermosa’s trajectory as a starting point for generating novel, testable theoretical hypotheses. Rather than claiming to have proven causal relationships, we invite future empirical research to investigate the following propositions, each derived directly from the patterns observed in this case.

### Hypothesis 1: the accountability-moderator hypothesis

The first hypothesis emerging from our analysis concerns the role of perceived accountability as a potential moderator of the relationship between institutional trauma and psychopathology. Drawing on Hermosa’s experience, where no form of accountability was present, we propose that when youth in juvenile detention perceive that the institution acknowledges its failures, provides redress for harm, and implements corrective actions, the negative mental health effects of institutional trauma may be significantly reduced. Conversely, when accountability is entirely absent, as in Hermosa’s case, institutional trauma may become a chronic stressor that compounds pre-existing vulnerabilities and accelerates antisocial outcomes. This hypothesis could be tested through longitudinal studies that measure youth perceptions of institutional accountability at multiple time points (intake, during detention, and at release) alongside validated psychopathology instruments assessing PTSD, depression, and conduct disorder symptoms. Such research would clarify whether accountability functions as a protective factor or merely a correlate of better institutional conditions.

### Hypothesis 2: the institutional trauma compounding hypothesis

The second hypothesis addresses the interaction between different sources of trauma across development. Hermosa’s trajectory suggests a possible compounding effect: early childhood maltreatment within the family may be exacerbated, rather than simply added to, by later institutional trauma experienced during detention. We hypothesize that youth exposed to both forms of trauma will exhibit greater severity of antisocial behavior and psychopathology than those exposed to only one, and that this effect may be more than additive, potentially multiplicative or synergistic. Testing this hypothesis would require a prospective cohort study comparing three groups of justice-involved youth: those with documented childhood maltreatment but no institutional trauma, those with institutional trauma but no prior maltreatment, and those exposed to both. Outcomes of interest would include recidivism rates, psychopathology scores, functional impairment, and, where feasible, neurobiological or psychophysiological measures. Such research would help determine whether interventions need to address cumulative trauma exposure in a staged or integrated manner.

### Hypothesis 3: the “schools of crime” hypothesis

The third hypothesis speaks directly to the criminogenic nature of certain juvenile detention environments. Based on Hermosa’s apparent skill consolidation during his incarceration at the Virgilio Guerrero Institute, we hypothesize that juvenile facilities characterized by high concentrations of antisocial peers, weak supervision, absent rehabilitative programming, and punitive rather than therapeutic staff practices function as “schools of crime.” In such environments, younger or less experienced offenders may learn specific criminal techniques, adopt cognitive distortions that justify violence, and expand antisocial networks—all of which may increase the severity and sophistication of offending upon release. This hypothesis could be tested through a mixed-methods comparative study of facilities that vary systematically along the dimensions described above. Quantitative outcomes would include post-release offending rates and offense severity measured via official records and self-report. Qualitative interviews with youth could illuminate the mechanisms through which skill transfer and antisocial modeling occur. If confirmed, this hypothesis would provide strong empirical support for reforming facility conditions and peer management practices as a violence prevention strategy.

## Clinical and research implications

These three hypotheses are not intended to be exhaustive but rather to demonstrate how a historical case, when analyzed through a theoretical lens, can generate empirically testable questions for the field. We acknowledge that documentary sources alone cannot confirm any of these propositions. Instead, we invite researchers to design studies that move beyond retrospective inference and directly test these hypotheses using rigorous empirical methods, including prospective longitudinal designs, validated instruments, and, where possible, neuroimaging or psychophysiological measures. By shifting from case-based description to hypothesis-driven investigation, the field can build an evidence base capable of informing juvenile justice reform in Latin America and beyond. Hermosa’s tragic trajectory cannot be changed, but the questions his case raises can be answered through systematic research—and those answers may prevent similar outcomes for future youth.

## Discussion

The case of Juan Fernando Hermosa illustrates the convergence of individual vulnerabilities and structural conditions in the emergence of juvenile serial homicide. His trajectory, marked by early animal cruelty, severe maltreatment, and escalating patterns of violence, reflects developmental models that link callous–unemotional traits and childhood trauma to persistent antisocial behavior (1, 2, 44). However, focusing solely on Hermosa’s psychopathology risks obscuring the broader Latin American context in which his crimes unfolded. Research on violence in the region highlights the role of structural inequality, weak institutions, and cultural legacies of violence in shaping youth delinquency and homicide rates (43). Rivera’s (45) empirical analysis of homicide trends between 1980 and 2010 further demonstrates that no single theory adequately explains Latin American violence; instead, a multi-theoretical approach that incorporates socioeconomic marginalization, institutional fragility, and cultural dynamics is required. Hermosa’s case exemplifies this intersection, where systemic failures in family, community, and institutional domains reinforced personal pathology.

The implications for prevention and intervention are significant. Hermosa exhibited clear warning signs, such as animal cruelty, gang involvement, and impulsivity, but these were left unaddressed in a context where preventive infrastructures were weak or non-existent. Evidence from developmental criminology emphasizes that school-based interventions, family support programs, and cognitive behavioral approaches can reduce delinquency and foster prosocial development (12, 46). The absence of such programs in Ecuador during the 1980s and 1990s represents a missed opportunity to intervene before Hermosa’s violence escalated to serial homicide.

More broadly, Latin American countries require preventive frameworks that combine individual-level psychological interventions with structural investments in education, mental health, and social protection, aligning with calls from regional violence research to address both proximal and distal risk factors simultaneously (43, 45). Comparisons with other juvenile offenders in Latin America reveal similar trajectories. Cases such as Pedro Pablo Nakada in Peru or Daniel Camargo Barbosa in Colombia and Ecuador demonstrate how childhood maltreatment, poverty, and marginalization intersect with psychopathic traits to produce extreme violence (47).

Hermosa’s incarceration at the Virgilio Guerrero Institute provides a stark illustration of institutional trauma, a concept central to this special issue. Unlike early childhood abuse within the family, institutional trauma refers to harm inflicted or exacerbated by systems designed to care for, confine, or rehabilitate youth. In Hermosa’s case, the institute did not merely fail to rehabilitate; it actively contributed to his worsening trajectory. He escaped, killed a police officer, and ultimately died under suspicious circumstances following recapture. These outcomes cannot be understood without examining the institutional context: absence of psychological treatment, exposure to violent peers, lack of educational programming, and, most critically, complete absence of accountability.

Accountability in juvenile justice settings can take multiple forms: procedural accountability (clear rules and consequences for staff misconduct), therapeutic accountability (providing evidence-based treatment and measuring outcomes), restorative accountability (acknowledging harm to victims and youth), and systemic accountability (external oversight and remediation when failures occur). None of these were present in Hermosa’s case. No staff member was sanctioned for the conditions that enabled escape. No investigation examined whether the institute’s neglect contributed to the subsequent homicide of a police officer. No apology or redress was offered to Hermosa’s victims or his family.

Contemporary research on institutional trauma emphasizes that perceived accountability can moderate the relationship between institutional harm and psychopathology. When youth believe that an institution acknowledges its failures and takes corrective action, mental health outcomes improve. Conversely, when accountability is absent, as in Hermosa’s case, institutional trauma becomes a chronic stressor that compounds pre-existing vulnerabilities. Hermosa entered the Virgilio Guerrero Institute with a history of childhood maltreatment; he left having killed again. This suggests that without accountability, the juvenile justice system risks becoming a trauma generator rather than a trauma healer.

For the special issue, this case offers a critical warning: even the most severe individual psychopathology cannot be understood in isolation from the institutional systems that shape it. Hermosa’s trajectory from “Child of Terror” to fugitive to alleged homicide victim was not inevitable. It was, in part, produced by an institution that failed to provide accountability. Latin American juvenile justice systems, often underfunded and punitive by default, must therefore prioritize the development of accountability mechanisms, external oversight, youth grievance procedures, staff training on trauma-informed care, and transparent error remediation, as essential components of any reform agenda.

The primary limitation of this theoretical study lies in the impossibility of accessing real data from a formal forensic psychological evaluation of Juan Fernando Hermosa, given that at the time the charges were brought, no expert assessments were conducted that would allow for an in-depth analysis of his psychological characteristics. Nevertheless, in this work, we have attempted to carry out a clinical analysis based on a critical and interpretative reading of the data found on the criminal activity of the “Child of Terror.” Nonetheless, key data are observed that help to understand how risk factors such as child abuse, neglect, violence against animals, and the absence of social rehabilitation processes in juvenile detention centers—as noted in this study—could lead a human being to end up in a criminal context such as that of Juan Fernando Hermosa.

On the other hand, it is interesting to reflect on what might have happened if Juan Fernando Hermosa had received all the necessary social support to face the difficulties that arose during his developmental process. For example, having a family that welcomed him, an educational institution that supported his cognitive development, psychological care for disruptive behaviors such as aggression or animal abuse, opportunities to participate in social groups—in short, with such ideal support, it is possible that the “Child of Terror” would have ended up as a person working for the benefit of humanity, rather than as someone who takes the lives of others. This case serves as a reflection for humanity, prompting us to consider what we are doing to ensure that future individuals can develop into beings who respect others, rather than becoming cannibals who may take from others without reflecting on the consequences of their actions.
